# Infectious burden and cellular immune impairment in higher-risk myelodysplastic syndromes with biallelic *TP53* alterations

**DOI:** 10.3389/fcimb.2026.1908881

**Published:** 2026-10-14

**Authors:** Binbin Lai, Xiao Yan, Lieguang Chen, Yi Wang, Lixia Sheng, Guifang Ouyang

**Affiliations:** Department of Hematology, The First Affiliated Hospital of Ningbo University, Ningbo, Zhejiang, China

**Keywords:** biallelic *TP53* alterations, cellular immune impairment, infection-related mortality, IPSS-M, myelodysplastic syndromes

## Abstract

**Background:**

Higher-risk myelodysplastic syndromes (MDS) are frequently complicated by severe infections, yet the impact of molecular subgroups on infection susceptibility remains poorly defined. Biallelic *TP53* (bi-*TP53*) alterations identify a high-risk molecular subset linked to adverse outcomes, but their contribution to infection incidence and infection-related mortality has not been systematically studied.

**Methods:**

We retrospectively analyzed 97 hospitalized higher-risk MDS patients (IPSS-R ≥4) identified through hospitalizations between June 2023 and April 2026. Patients were grouped by *TP53* allelic status, and infection-related outcomes were compared. Clinically significant infections were defined as suspected or documented events requiring inpatient management and/or intravenous anti-infective therapy. Outcomes included infection incidence, recurrent infection, anti-infective escalation, baseline lymphocyte subsets, overall survival, and composite event-free survival (cEFS; infection-related death or AML transformation). Cox regression evaluated cEFS, and Firth penalized logistic regression assessed infection-related death. IPSS-M risk category was also analyzed.

**Results:**

Among 97 patients, 31 had bi-*TP53* alterations. During a median follow-up of 0.8 years, 59 patients (60.8%) developed clinically significant infections and 27 (27.8%) died from infection. Compared with non-bi-*TP53* patients, bi-*TP53* patients had higher rates of infection (87.1% vs. 48.5%), recurrent infection (54.8% vs. 30.3%), infection within 100 days after diagnosis (77.4% vs. 28.8%), and infection within 1 year after diagnosis (83.9% vs. 39.4%; all P<0.05). Among infected patients, bi-*TP53* alterations were associated with more severe infection, greater anti-infective treatment escalation (85.2% vs. 31.3%), more frequent antifungal therapy use (74.1% vs. 15.6%), and higher infection-related mortality (85.2% vs. 12.5%; all P<0.001). Baseline neutrophil, hemoglobin, and platelet counts were comparable between groups, whereas CD3+ T-cell, CD4+ T-cell, CD8+ T-cell, and NK-cell counts were significantly lower in bi-*TP53* patients. In multivariable analysis, bi-*TP53* alterations remained associated with shorter cEFS (HR = 7.56, 95% CI: 2.79–20.46) and infection-related death among infected patients (OR = 7.52, 95% CI: 1.15–49.37).

**Conclusions:**

Bi-*TP53* alterations were associated with greater infectious burden, lower circulating T-cell and NK-cell counts, and higher infection-related mortality in hospitalized patients with higher-risk MDS. These observational findings do not establish causality or independence from neutropenia. Prospective multicenter studies should assess their value for infection risk stratification and supportive-care planning.

## Introduction

Myelodysplastic syndromes/neoplasms (MDS) are heterogeneous clonal hematopoietic stem cell disorders characterized by ineffective hematopoiesis, morphologic dysplasia, peripheral cytopenias, and a variable risk of progression to acute myeloid leukemia (AML) (Cazzola, 2020). Patients with higher-risk MDS, commonly defined by an IPSS-R score ≥4, have particularly poor outcomes, with a median overall survival of approximately 16–21 months (Greenberg et al., 2012). Although leukemic transformation remains a major cause of disease-related mortality, infectious complications are also frequent and often fatal in this population, accounting for a substantial proportion of deaths despite advances in supportive care and disease-directed therapy (Nachtkamp et al., 2016). Traditionally, infection risk in MDS has been attributed mainly to quantitative cytopenias, especially neutropenia, as well as treatment-related immunosuppression (Houtman et al., 2025). However, whether infectious vulnerability in MDS is shaped by molecular disease architecture, rather than only by conventional hematologic and treatment-related factors, remains largely unexplored.

Biallelic *TP53* inactivation, also referred to as multi-hit *TP53* alteration or bi-*TP53*, represents one of the most adverse molecular lesions in MDS (Bernard et al., 2020). This genetic state reflects loss of normal *TP53* function through combined mutational, cytogenetic, or copy-number mechanisms and is strongly associated with genomic instability, complex karyotype, treatment resistance, rapid disease progression, and dismal survival (Bernard et al., 2020). Emerging evidence has suggested a potential association between *TP53*-altered myeloid disease and infectious complications, including pulmonary infection, invasive fungal infection, and infection-related mortality (Chen et al., 2023; Marvin-Peek et al., 2023). Nevertheless, the infectious burden specifically associated with bi-*TP53* alterations in strictly defined higher-risk MDS remains poorly characterized.

The International Prognostic Scoring System-Molecular (IPSS-M) integrates clinical, cytogenetic, and molecular abnormalities to refine risk stratification in MDS (Bernard et al., 2022). By incorporating molecular lesions, including *TP53* allelic status, IPSS-M better captures adverse disease biology than conventional scoring systems and identifies patients with very-high-risk disease who have inferior survival and a high probability of AML transformation (Bernard et al., 2022). However, whether IPSS-M-defined molecular risk is also associated with infectious complications has rarely been investigated. Therefore, evaluating infection-related outcomes in relation to both bi-*TP53* status and IPSS-M risk classification may help clarify whether molecularly defined adverse disease biology is linked to infectious vulnerability in higher-risk MDS.

Here, we investigated infection-related outcomes in a cohort of hospitalized patients with higher-risk MDS, focusing on bi-*TP53* alterations. We assessed infection burden, severity, and infection-related mortality in relation to *TP53* allelic status and IPSS-M risk classification. We also compared baseline circulating lymphocyte subsets to examine the association between bi-*TP53* status, cellular immune profiles, and infectious outcomes.

## Materials and methods

### Study design and participants

This single-center retrospective cohort study identified patients through MDS-related hospitalizations between June 1, 2023 and April 30, 2026. This interval defined cohort identification, whereas individual follow-up began at MDS diagnosis. The administrative database closure date was April 30, 2026. Diagnosis and longitudinal records were retrospectively retrieved from the institutional electronic medical record system. MDS was diagnosed according to established classification criteria (Arber et al., 2022), and higher-risk MDS was defined as a Revised International Prognostic Scoring System (IPSS-R) score ≥4 (Greenberg et al., 2012).

Patients were eligible for inclusion in the analysis if they had higher-risk MDS according to the prespecified IPSS-R definition; had available baseline hematologic, bone marrow, cytogenetic, and molecular data; and had traceable information on infection events, disease progression, and survival outcomes.

Patients were excluded if they had lower-risk MDS, defined as an IPSS-R score <4; had a history of hematopoietic stem cell transplantation (HSCT); had MDS/myeloproliferative neoplasm overlap syndrome or MDS with overt bone marrow fibrosis; had an uncertain diagnosis or a final diagnosis of another hematologic disorder, such as aplastic anemia, acute myeloid leukemia, or immune thrombocytopenia; had incomplete baseline, infection-related, or follow-up outcome data; or had special comorbid conditions likely to substantially confound infection assessment or survival analysis, including active second malignancy, severe primary immunodeficiency, or other complex comorbidities requiring separate clinical interpretation. The patient selection process is shown in **Figure 1**. The study was approved by the Ethics Committee of the First Affiliated Hospital of Ningbo University (Approval No. 2026-R216-01), and the requirement for informed consent was waived owing to the retrospective nature of the study.

**Figure 1:**
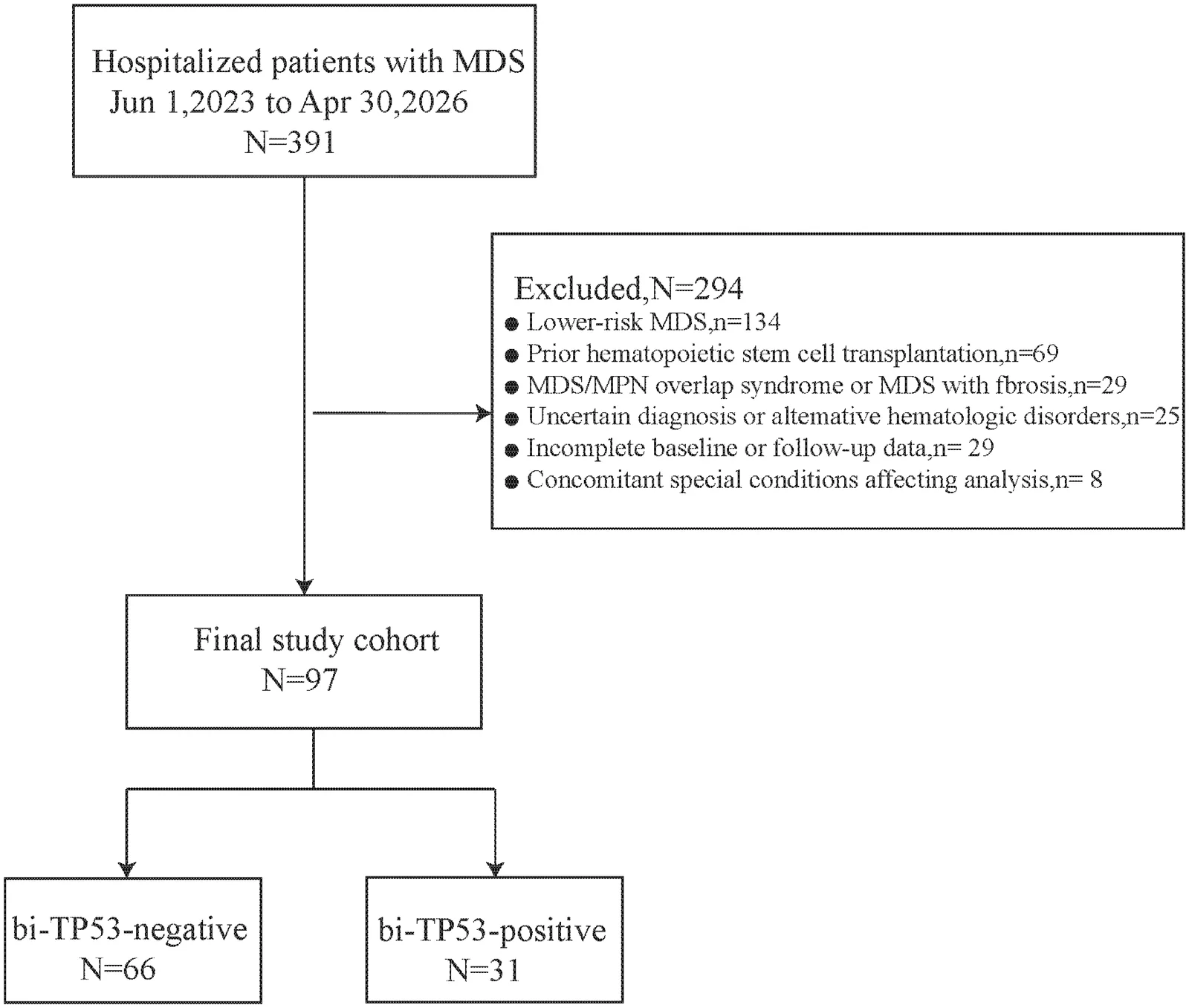
Patient selection flow diagram. Flow diagram of the retrospective cohort derivation.

### Data collection and variable definitions

Clinical data were retrospectively extracted from the electronic medical record system for all eligible hospitalizations and traceable follow-up encounters within the institution. Variables collected for analysis included baseline demographic and clinical characteristics, comorbidities, hematologic and immune-cell parameters, bone marrow findings, cytogenetic and molecular results, prognostic risk categories, treatment exposure, infection-related variables, anti-infective treatment, AML transformation, death, and cause of death. Individual follow-up duration was calculated from MDS diagnosis to death or the last documented follow-up before database closure. The median and interquartile range were calculated directly from these intervals, without reverse Kaplan–Meier estimation. The maximum reported follow-up was 4299 days (approximately 141.2 months), reflecting diagnoses that predated the cohort-identification period.

Baseline variables were defined as data obtained at the time of MDS diagnosis. These included age, sex, diabetes mellitus, Myelodysplastic Syndrome-specific Comorbidity Index (MDS-CI), absolute neutrophil count (ANC), hemoglobin level, platelet count, serum ferritin, transfusion dependence, bone marrow blast percentage, karyotype risk category according to the IPSS-R cytogenetic risk classification, IPSS-R risk category, Molecular International Prognostic Scoring System (IPSS-M) risk category, and molecular findings. Baseline immune measurements included absolute counts of CD3+ T cells, CD4+ T cells, CD8+ T cells, CD19+ B cells, and natural killer (NK) cells, the absolute lymphocyte count, and the CD4/CD8 ratio. These measurements were obtained at diagnosis, before documented infection. Lymphocyte subsets were measured using a DxFLEX flow cytometer (Beckman Coulter, USA) and commercial antibody reagents (Jiangxi Derps Biotechnology Co., Ltd., China). Absolute counts of CD3+, CD4+, CD8+, CD19+, and CD56+ cells were determined by flow cytometry using counting beads and expressed as cells/μL.

Patients were classified into two groups according to *TP53* allelic status: the bi-*TP53* group, defined by the presence of biallelic *TP53* alterations, and the non-bi-*TP53* group, defined by the absence of biallelic *TP53* alterations. Molecular alterations were identified by targeted next-generation sequencing of bone marrow aspirates collected at diagnosis. Sequencing used the DNBSEQ-T1+ platform (MGI Tech, Shenzhen, China), with coverage of all coding exons of *TP53*. Cytogenetic abnormalities were assessed using conventional karyotyping and/or fluorescence *in situ* hybridization when available. Bi-*TP53* alterations were defined according to established criteria as: two or more distinct *TP53* mutations with a variant allele frequency (VAF) ≥10%; or a single *TP53* mutation accompanied by one of the following features: deletion involving the *TP53* locus at 17p13.1, VAF >50%, or copy-neutral loss of heterozygosity involving the 17p *TP53* locus. In the absence of loss-of-heterozygosity information, a single *TP53* mutation occurring in the context of complex karyotype was considered consistent with bi-*TP53* alteration (Arber et al., 2022). For IPSS-M analysis, the very-high-risk (VH) category was analyzed as a key molecular risk group and compared with non-very-high-risk categories. Treatment was categorized as best supportive care, hypomethylating agent (HMA) therapy, or HMA-based combination therapy. HMA monotherapy consisted of either azacitidine (75 mg/m² for 7 days) or decitabine (15 mg/m² for 5 days). HMA-based combination therapy consisted of either azacitidine or decitabine combined with cytarabine (30 mg for 7 days) or venetoclax (100 mg on day 1, 200 mg on day 2, and 400 mg on days 3–15). Treatment cycles were scheduled every 28 days. No corticosteroid or other immunosuppressive-agent exposure was documented as part of the MDS-directed treatment regimens.

### Definition of infection events

Infection events were adjudicated using inpatient clinical notes, physician diagnoses, imaging findings, microbiologic evidence, anti-infective treatment records, and discharge diagnoses. Clinically significant infection events were defined as clinically suspected or documented infections requiring inpatient management and/or intravenous anti-infective therapy. Microbiologic confirmation was not mandatory when compatible clinical, laboratory, radiologic, and treatment evidence supported an infectious diagnosis. Mild, outpatient, or self-limited infections that did not require inpatient management or intravenous anti-infective therapy were not included in the primary analysis. Infection severity was retrospectively graded according to the National Cancer Institute Common Terminology Criteria for Adverse Events (CTCAE), version 6.0. CTCAE grade 5 infection was defined as infection-related death, determined when an active infection was judged to be the primary or a major contributing cause of death based on medical records, discharge diagnoses, and documented cause of death. Febrile neutropenia (FN) was defined as fever, either a single temperature ≥38.3 °C or a sustained temperature ≥38.0 °C for more than 1 hour, accompanied by an absolute neutrophil count <0.5 × 10^9^/L or expected to decrease to <0.5 × 10^9^/L within 48 hours. Recurrent infection was defined as at least two clinically significant infection episodes, irrespective of whether the same pathogen was identified. This definition did not establish microbiologically confirmed relapse or reinfection with the same organism.

Composite event-free survival (cEFS) was prespecified as the time from MDS diagnosis to the first occurrence of infection-related death or transformation to AML, whichever occurred first. Patients without either event were censored at the last follow-up. AML transformation ended event-free follow-up for cEFS but did not itself end follow-up for OS. Overall survival (OS) was defined as the time from MDS diagnosis to death from any cause, with patients alive at the last follow-up censored.

### Statistical analysis

Continuous variables are presented as mean ± standard deviation or median with interquartile range, depending on distribution, and categorical variables are presented as frequencies and percentages. Between-group comparisons were performed using Student’s *t* test, the Mann–Whitney U test, the chi-square test, or Fisher’s exact test, as appropriate. Comparative analyses were first performed between patients with and without infection, then between patients who died from infection and those who did not, and further, among infected patients only, between those with and without infection-related death.

Spearman correlation analysis described relationships among clinical, disease-risk, molecular, immune-cell, and infection-related variables. The matrix informed assessment of overlap and potential collinearity during model construction but did not establish statistical independence. Cox model covariates were selected using univariable associations (P < 0.05), clinical relevance, and assessment of potential collinearity. Febrile neutropenia was excluded after assessment of its correlation with baseline ANC. Univariable logistic regression explored associations with infection occurrence. Kaplan–Meier analysis estimated OS and cEFS, with log-rank tests used for group comparisons. Cox proportional hazards regression evaluated associations with cEFS, reported as hazard ratios (HRs) with 95% confidence intervals (CIs). Analyses involving infection status were exploratory because infection was determined during follow-up rather than at baseline.

Among infected patients, Firth penalized logistic regression evaluated associations with infection-related death, given the limited number of events. Results are reported as odds ratios (ORs) with 95% CIs. Variables with univariable P < 0.01 were considered for the multivariable Firth model. No adjustment for multiple comparisons was performed because the analyses were exploratory. Analyses used R version 4.5.0 and the logistf package for Firth regression. Two-sided P values <0.05 were considered statistically significant.

## Results

### Study cohort and overall characteristics

During the study period, 391 hospitalized patients with MDS were screened. After exclusion of 294 patients, 97 patients were included in the final analysis (**Figure 1**). The mean age at diagnosis was 69.8 ± 10.0 years, and 62 patients (63.9%) were male. The median follow-up was 0.8 (0.4–1.7) years. During follow-up, 59 patients (60.8%) developed infection and 27 patients (27.8%) had infection-related death. According to IPSS-R risk stratification, 37 patients (38.1%) were classified as intermediate risk, 33 (34.0%) as high risk, and 27 (27.8%) as very high risk. According to IPSS-M risk stratification, 44 patients (45.4%) were classified as VH. Overall, 31 patients (32.0%) had bi-*TP53* alterations.

### Characteristics of patients with infection and infection-related death

Compared with patients without infection, infected patients differed mainly in disease-risk and molecular features. The proportion of patients with bi-*TP53* alterations was higher among infected patients than among non-infected patients (27/59, 45.8% vs. 4/38, 10.5%; P<0.001). Karyotype risk distribution, IPSS-R category, and IPSS-M category also differed significantly between infected and non-infected patients, with P values of 0.009, 0.006, and <0.001, respectively. Among infected patients, 37 (62.7%) were IPSS-M VH and 23 (39.0%) were IPSS-R very high risk.

Infected patients had higher serum ferritin levels than non-infected patients (P = 0.039). Lymphocyte subset measurements also differed between infected and non-infected patients, including CD3 (P = 0.005), CD4 (P<0.001), CD8 (P = 0.011), and NK cells (P = 0.001). In contrast, sex, diabetes mellitus, baseline ANC <0.8×10^9/L, hemoglobin <8 g/dL, platelets <50×10^9/L, transfusion dependence, bone marrow blast category, and MDS-CI distribution were not significantly different between the two groups.

Among the 59 infected patients, 27 had infection-related death and 32 did not. Within the infected subgroup, patients with infection-related death had shorter follow-up and a higher concentration of adverse disease features. Bi-*TP53* alterations were present in 23/27 (85.2%) patients with infection-related death and was significantly more frequent than in infected patients without infection-related death (P<0.001). Very poor karyotype was observed in 21/27 (77.8%) patients with infection-related death (P<0.001), IPSS-R very high risk in 17/27 (63.0%) (P<0.001), and IPSS-M VH in 25/27 (92.6%) (P<0.001). Treatment distribution also differed within the infected subgroup (P<0.001), with supportive care recorded in 20/27 (74.1%) patients with infection-related death. Age, baseline blood counts, serum ferritin, bone marrow blast category, MDS-CI, lymphocyte subsets, and infection site distribution did not significantly differ between infected patients with and without infection-related death (**Table 1**).

**Table 1:** Baseline clinical, genetic, and immunologic characteristics according to infection status and infection-related death.

Characteristics	Included cohort	Infection	P	Infection led to death	P*	P**
Total, N	97	59	—	27	—	—
Baseline characteristics						
Age at diagnosis, years	69.8 ± 10.0	71.3 ± 9.8	0.076	73.1 ± 7.7	0.045	0.194
Follow-up time, years	0.8 (0.4–1.7)	0.7 (0.3–1.5)	0.25	0.4 (0.2–0.6)	<0.001	<0.001
Sex, N (%)						
Male	62 (63.9)	40 (67.8)	0.439	15 (55.6)	0.407	0.117
Female	35 (36.1)	19 (32.2)		12 (44.4)		
Diabetes mellitus, N (%)	21 (21.6)	14 (23.7)	0.714	8 (29.6)	0.363	0.502
ANC <0.8×10^9/L, N (%)	41 (42.3)	23 (39.0)	0.545	9 (33.3)	0.38	0.583
Hemoglobin <8 g/dL, N (%)	60 (61.9)	37 (62.7)	0.998	18 (66.7)	0.709	0.759
Platelets <50×10^9/L, N (%)	37 (38.1)	22 (37.3)	0.998	11 (40.7)	0.925	0.815
Serum ferritin	346.2 (210.3–560.3)	360.7 (254.4–623.2)	0.039	434.9 (306.4–630.0)	0.029	0.146
Transfusion dependence, N (%)						
No	70 (72.2)	42 (71.2)	0.971	19 (70.4)	1	1
Yes	27 (27.8)	17 (28.8)		8 (29.6)		
Bi-TP53, N (%)	31 (32.0)	27 (45.8)	<0.001	23 (85.2)	<0.001	<0.001
Karyotype category, N (%)						
Very good	2 (2.1)	1 (1.7)	0.009	1 (3.7)	<0.001	<0.001
Good	53 (54.6)	25 (42.4)		3 (11.1)		
Intermediate	11 (11.3)	6 (10.2)		1 (3.7)		
Poor	2 (2.1)	2 (3.4)		1 (3.7)		
Very poor	29 (29.9)	25 (42.4)		21 (77.8)		
Bone marrow blasts category, N (%)						
≤5%	43 (44.3)	25 (42.4)	0.839	12 (44.4)	0.962	0.841
>5–<10%	34 (35.1)	22 (37.3)		9 (33.3)		
≥10%	20 (20.6)	12 (20.3)		6 (22.2)		
MDS-CI, N (%)						
0	48 (49.5)	27 (45.8)	0.791	11 (40.7)	0.437	0.56
1	35 (36.1)	23 (39.0)		11 (40.7)		
2	12 (12.4)	8 (13.6)		5 (18.5)		
≥3	2 (2.1)	1 (1.7)		0 (0.0)		
IPSS-R category, N (%)						
Intermediate	37 (38.1)	17 (28.8)	0.006	2 (7.4)	<0.001	<0.001
High	33 (34.0)	19 (32.2)		8 (29.6)		
Very high	27 (27.8)	23 (39.0)		17 (63.0)		
IPSS-M category, N (%)						
Low	2 (2.1)	0 (0.0)	<0.001	0 (0.0)	<0.001	<0.001
Moderate low	13 (13.4)	7 (11.9)		0 (0.0)		
Moderate high	16 (16.5)	6 (10.2)		0 (0.0)		
High	22 (22.7)	9 (15.3)		2 (7.4)		
Very high	44 (45.4)	37 (62.7)		25 (92.6)		
Immune cell indicators						
CD3	667.8 ± 378.3	582.4 ± 354.9	0.005	547.1 ± 313.3	0.05	0.487
CD4	343.0 (193.0–552.0)	214.0 (148.5–379.5)	<0.001	193.0 (121.0–286.0)	<0.001	0.057
CD8	203.3 ± 140.4	174.4 ± 127.1	0.011	142.9 ± 102.3	0.008	0.081
CD4/CD8 ratio	1.8 (1.0–2.5)	1.9 (1.1–2.5)	0.377	1.9 (1.5–2.6)	0.113	0.183
CD19	34.0 (16.0–55.0)	41.0 (16.0–82.5)	0.534	45.0 (27.5–82.5)	0.1	0.112
NK	88.0 (54.0–194.0)	78.0 (41.5–112.0)	0.001	70.0 (38.0–107.5)	0.031	0.469
Absolute lymphocyte count, cells/μL	820.0 (460.0–1097.0)	669.0 (420.0–1083.0)	0.113	846.0 (454.0–1398.0)	0.606	0.173
Treatment, N (%)						
Supportive care	44 (45.4)	28 (47.5)	0.537	20 (74.1)	<0.001	<0.001
HMA	32 (33.0)	17 (28.8)		2 (7.4)		
HMA+	21 (21.6)	14 (23.7)		5 (18.5)		

P: comparison between patients with infection and patients without infection.

P*: univariable comparison between patients with infection-related death and all other patients.

P**: among infected patients only, comparison between infection-related death and infected patients without infection-related death.

Abbreviations: IPSS-R, Revised International Prognostic Scoring System; IPSS-M, Molecular International Prognostic Scoring System; MDS-CI, Myelodysplastic syndrome-specific comorbidity index; ANC, absolute neutrophil count; FN, febrile neutropenia; CTCAE, Common Terminology Criteria for Adverse Events; SD, standard deviation; IQR, interquartile range.

### Disease features and immune cell profiles according to bi-*TP53* status

When patients were stratified by bi-*TP53* status, 66 were in the non-bi-*TP53* group and 31 were in the bi-*TP53* group. Age and sex distribution were similar between groups, whereas follow-up was shorter in the bi-*TP53* group than in the non-bi-*TP53* group [0.30 (0.21–0.48) vs. 1.16 (0.67–2.81) years; P<0.001].

Patients with bi-*TP53* alterations had fewer co-mutations [0.00 (0.00–1.00) vs. 2.00 (1.00–3.00); P<0.001] and higher serum ferritin levels [448.40 (306.35–713.05) vs. 285.40 (180.95–487.60); P = 0.005] (**Table 2**). *TP53* was not the only molecular abnormality detected in the bi-*TP53* group; accompanying alterations are summarized as co-mutation counts in **Table 2**. Cytogenetic findings are summarized using IPSS-R risk categories in **Table 1**, rather than individual chromosomal lesions.

**Table 2:** Clinical and infection-related characteristics according to bi-TP53 status.

Characteristics	Non bi-TP53 group (N = 66)	Bi-TP53 group (N = 31)	P value
**Overall cohort**	**66**	**31**	**-**
Disease biology and baseline cytopenias			
Age at diagnosis, years	68.94 ± 10.63	71.77 ± 8.34	0.194
Sex, N (%)			
Female	23 (34.8)	12 (38.7)	0.887
Male	43 (65.2)	19 (61.3)	
Follow-up time, years	1.16 (0.67–2.81)	0.30 (0.21–0.48)	<0.001
Baseline ANC <0.8×10^9/L, N (%)	30 (45.5)	11 (35.5)	0.48
Hemoglobin <8 g/dL, N (%)	39 (59.1)	21 (67.7)	0.553
Platelets <50×10^9/L, N (%)	25 (37.9)	12 (38.7)	1
Serum ferritin	285.40 (180.95–487.60)	448.40 (306.35–713.05)	0.005
Number of co-mutations	2.00 (1.00–3.00)	0.00 (0.00–1.00)	<0.001
Bone marrow blasts category, N (%)			
≤5%	30 (45.5)	13 (41.9)	0.875
>5% to <10%	22 (33.3)	12 (38.7)	
≥10%	14 (21.2)	6 (19.4)	
IPSS-R category, N (%)			
INT	36 (54.5)	1 (3.2)	<0.001
H	24 (36.4)	9 (29.0)	
VH	6 (9.1)	21 (67.7)	
IPSS-M category, N (%)			
L	2 (3.0)	0 (0.0)	<0.001
ML	13 (19.7)	0 (0.0)	
MH	16 (24.2)	0 (0.0)	
H	22 (33.3)	0 (0.0)	
VH	13 (19.7)	31 (100.0)	
Cellular immune profile			
CD3	729.05 ± 387.07	537.29 ± 327.76	0.019
CD4	398.50 (212.75–639.00)	193.00 (121.00–240.50)	<0.001
CD8	237.38 ± 144.27	130.61 ± 99.74	<0.001
CD4/CD8 ratio	1.36 (1.01–2.50)	2.20 (1.26–2.75)	0.091
CD19	34.00 (12.75–55.00)	44.00 (22.50–69.00)	0.233
NK	101.00 (62.50–260.00)	54.00 (38.00–88.00)	<0.001
Absolute lymphocyte count, cells/μL	667.50 (460.00–1084.00)	1000.00 (466.50–1398.00)	0.396
Infection burden and clinical outcome			
Recurrent infection (≥2 episodes), N (%)	20 (30.3)	17 (54.8)	0.036
Infection rate, episodes/person-year	0.00 (0.00–1.17)	7.93 (3.59–9.74)	<0.001
Infection within 100 days after diagnosis, N (%)	19 (28.8)	24 (77.4)	<0.001
Infection within 1 year after diagnosis, N (%)	26 (39.4)	26 (83.9)	<0.001
AML transformation, N (%)	15 (22.7)	3 (9.7)	0.207
Death, N (%)	21 (31.8)	26 (83.9)	<0.001
Death cause, N (%)			
Alive	45 (68.2)	5 (16.1)	<0.001
Primary disease death	16 (24.2)	3 (9.7)	
Infection-related death	4 (6.1)	23 (74.2)	
Other death	1 (1.5)	0 (0.0)	
Total infected patients, N (%)	32 (48.5)	27 (87.1)	<0.001
**Infection-related characteristics among infected patients only**	**n=32**	**n=27**	
ANC at infection/baseline ANC, ×10^9/L	0.85 (0.47–1.73)	1.10 (0.65–1.80)	0.12
CRP at infection	32.67 (18.06–69.52)	56.40 (25.60–94.55)	0.142
Maximum CRP during infection	83.90 ± 67.17	112.30 ± 76.87	0.135
Infection-related death, N (%)	4 (12.5)	23 (85.2)	<0.001
FN, N (%)	11 (34.4)	14 (51.9)	0.176
CTCAE grade, N (%)			
Grade 3	27 (84.4)	4 (14.8)	<0.001
Grade 4	1 (3.1)	0 (0.0)	
Grade 5	4 (12.5)	23 (85.2)	
Antibiotic level at infection, N (%)			
IV standard antibiotics	3 (9.4)	1 (3.7)	<0.001
IV advanced/combination antibiotics	24 (75.0)	6 (22.2)	
Antifungal added	5 (15.6)	20 (74.1)	
Microbiologically confirmed recurrence of the same organism, N (%)	2 (6.3)	3 (11.1)	0.649
Antibiotic treatment upgrade at infection, N (%)	10 (31.3)	23 (85.2)	<0.001

Infection-related variables were analyzed among infected patients only. FN, febrile neutropenia.

IPSS-R , Revised International Prognostic Scoring System; IPSS-M, Molecular International Prognostic Scoring System; MDS-CI, Myelodysplastic syndrome-specific comorbidity index; ANC, absolute neutrophil count; CRP , C-reactive protein; OS , overall survival; AML , acute myeloid leukemia.

Bold values indicate the sample sizes of the corresponding study groups or analysis subsets.

Compared with patients in the non-bi-*TP53* group, those in the bi-*TP53* group had lower CD3+ T-cell counts (537.29 ± 327.76 vs. 729.05 ± 387.07; P = 0.019), CD4+ T-cell counts [193.00 (121.00–240.50) vs. 398.50 (212.75–639.00); P<0.001], CD8+ T-cell counts (130.61 ± 99.74 vs. 237.38 ± 144.27; P<0.001), and NK-cell counts [54.00 (38.00–88.00) vs. 101.00 (62.50–260.00); P<0.001]; all counts are expressed as cells/μL (**Table 2**).

### Infection burden and severity according to bi-*TP53* status

Infection-related outcomes differed markedly according to bi-*TP53* status. Infection occurred in 27 of 31 patients (87.1%) in the bi-*TP53* group and 32 of 66 patients (48.5%) in the non-bi-*TP53* group (P<0.001). Recurrent infection, defined as two or more infectious episodes, was more frequent in the bi-*TP53* group than in the non-bi-*TP53* group (17/31, 54.8% vs. 20/66, 30.3%; P = 0.036). Infection incidence density was also higher in the bi-*TP53* group [7.93 (3.59–9.74) vs. 0.00 (0.00–1.17) episodes/person-year; P<0.001]. Infection within 100 days after diagnosis occurred in 24 of 31 patients (77.4%) in the bi-*TP53* group and 19 of 66 patients (28.8%) in the non-bi-*TP53* group (P<0.001), while infection within 1 year occurred in 26 of 31 (83.9%) and 26 of 66 (39.4%) patients, respectively (P<0.001). At the infection-episode level, 146 infection episodes were recorded among the 59 infected patients (**Supplementary Table S1**).

Among infected patients, infection severity and anti-infective treatment intensity also differed between groups. In the non-bi-*TP53* group, most infected patients had CTCAE grade 3 infection (27/32, 84.4%), whereas grade 5 infection occurred in 4 of 32 patients (12.5%). In contrast, in the bi-*TP53* group, grade 5 infection occurred in 23 of 27 infected patients (85.2%), while grade 3 infection was recorded in 4 of 27 patients (14.8%); no grade 4 infection was recorded in the bi-*TP53* group. The CTCAE grade distribution differed significantly between the two groups (P<0.001) (**Table 2**).

Anti-infective treatment intensity was also higher among infected patients with bi-*TP53* alterations. Antifungal therapy was added in 20 of 27 infected patients (74.1%) in the bi-*TP53* group, compared with 5 of 32 infected patients (15.6%) in the non-bi-*TP53* group. Antibiotic treatment upgrade at infection was recorded in 23 of 27 infected patients (85.2%) in the bi-*TP53* group and 10 of 32 infected patients (31.3%) in the non-bi-*TP53* group (P<0.001).

Among infected patients, infection-related death occurred in 23 of 27 patients (85.2%) in the bi-*TP53* group and 4 of 32 patients (12.5%) in the non-bi-*TP53* group (P<0.001). In the overall cohort, death occurred in 26 of 31 patients (83.9%) in the bi-*TP53* group and 21 of 66 patients (31.8%) in the non-bi-*TP53* group (P<0.001). AML transformation occurred in 3 of 31 patients (9.7%) in the bi-*TP53* group and 15 of 66 patients (22.7%) in the non-bi-*TP53* group, without a statistically significant difference (P = 0.207). MicrobiolMicrobiologically confirmed recurrence of the same organism was observed in five patients, including two patients in the non-bi-*TP53* group (2/32, 6.3%) and three patients in the bi-*TP53* group (3/27, 11.1%; P = 0.649; **Table 2**).

### Univariable infection analysis

Spearman correlation analysis described relationships among the measured variables (**Figure 2A**). The matrix was exploratory, particularly because IPSS-R, IPSS-M, cytogenetic risk, and *TP53* allelic status share prognostic information. In univariable logistic regression, IPSS-R level, IPSS-M very-high-risk status, and bi-*TP53* alterations were associated with higher odds of infection. Lower CD3+, CD4+, and CD8+ T-cell counts and NK-cell counts were also associated with infection occurrence (**Figure 2B**).

**Figure 2:**
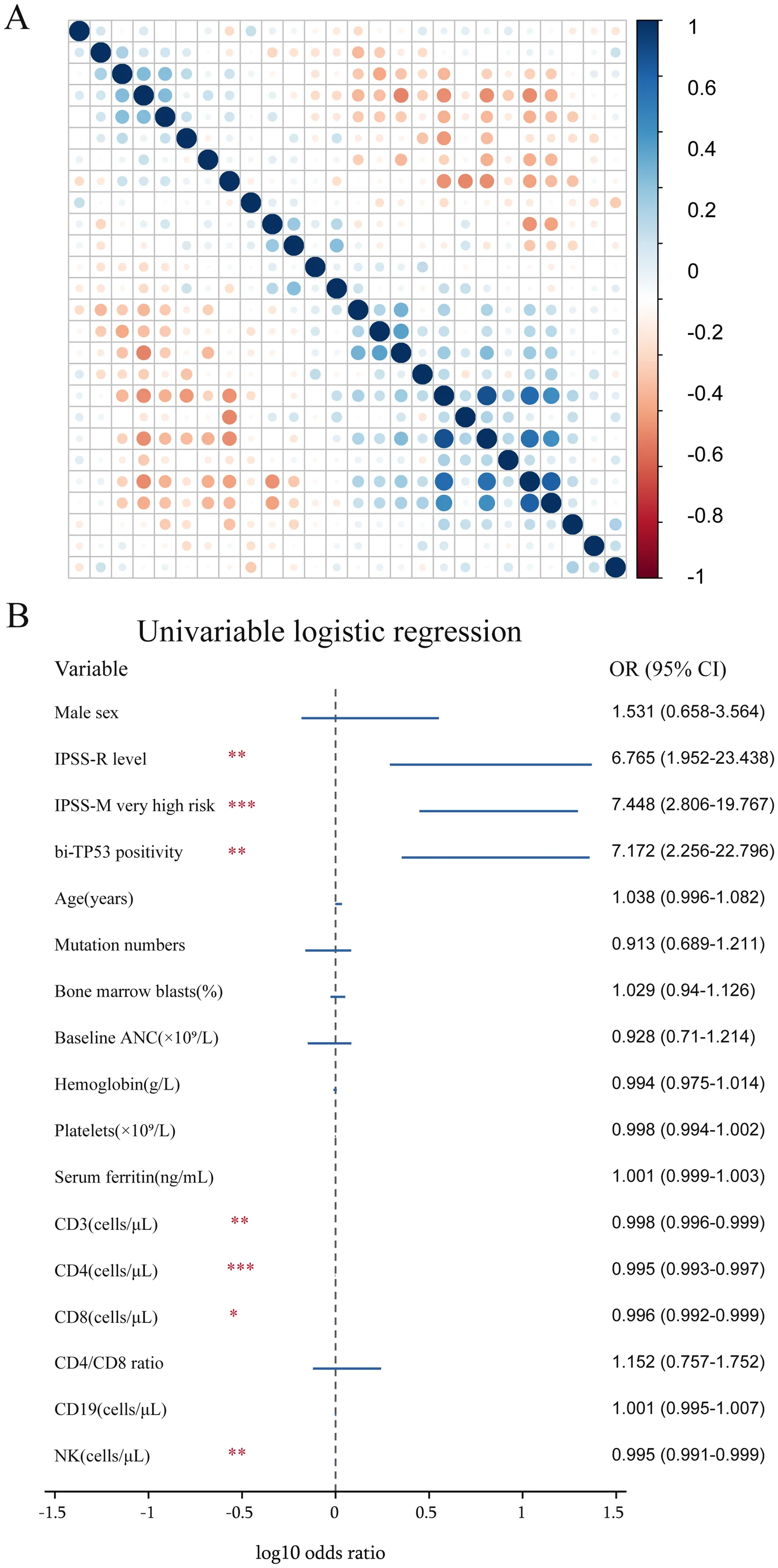
Factors associated with infection occurrence. **(A)** Exploratory Spearman correlation matrix of clinical, molecular, prognostic, immune-cell, and infection-related variables. Correlations describe relationships between measured variables and do not establish independent effects. **(B)** Univariable logistic regression for infection occurrence. Points represent odds ratios (ORs), and horizontal bars represent 95% confidence intervals (CIs), displayed on a log10 OR axis. For continuous variables, ORs correspond to increases of 1 year in age, one mutation, one percentage point in bone marrow blasts, 1 × 10^9^/L in ANC or platelet count, 1 g/L in hemoglobin, or 1 ng/mL in serum ferritin. ORs for CD3+, CD4+, CD8+, CD19+, and NK-cell counts correspond to an increase of 1 cell/μL; the CD4/CD8 ratio is expressed per one-unit increase. *P < 0.05, **P < 0.01, ***P < 0.001. P values were not adjusted for multiple comparisons.

### Overall survival and composite event-free survival

Kaplan–Meier analysis yielded a median OS of 16.9 months and a median cEFS of 15.6 months (**Figure 3A**). There were 47 deaths from any cause and 50 censored observations in the OS analysis. The cEFS analysis included 45 first events, comprising 27 infection-related deaths and 18 AML transformations, with 52 censored observations. Patients with bi-*TP53* alterations had shorter cEFS than those without biallelic alterations (log-rank P<0.001; **Figure 3B**). cEFS was also shorter in the IPSS-M very-high-risk group (log-rank P<0.001; **Figure 3C**). Within that risk group, patients with bi-*TP53* alterations had shorter cEFS than those without biallelic alterations (log-rank P<0.001; **Figure 3D**). An exploratory comparison showed shorter cEFS among patients with infections documented during follow-up (log-rank P<0.001; **Figure 3E**). This comparison was susceptible to time-related bias and overlap between infection status and the infection-related component of cEFS.

**Figure 3:**
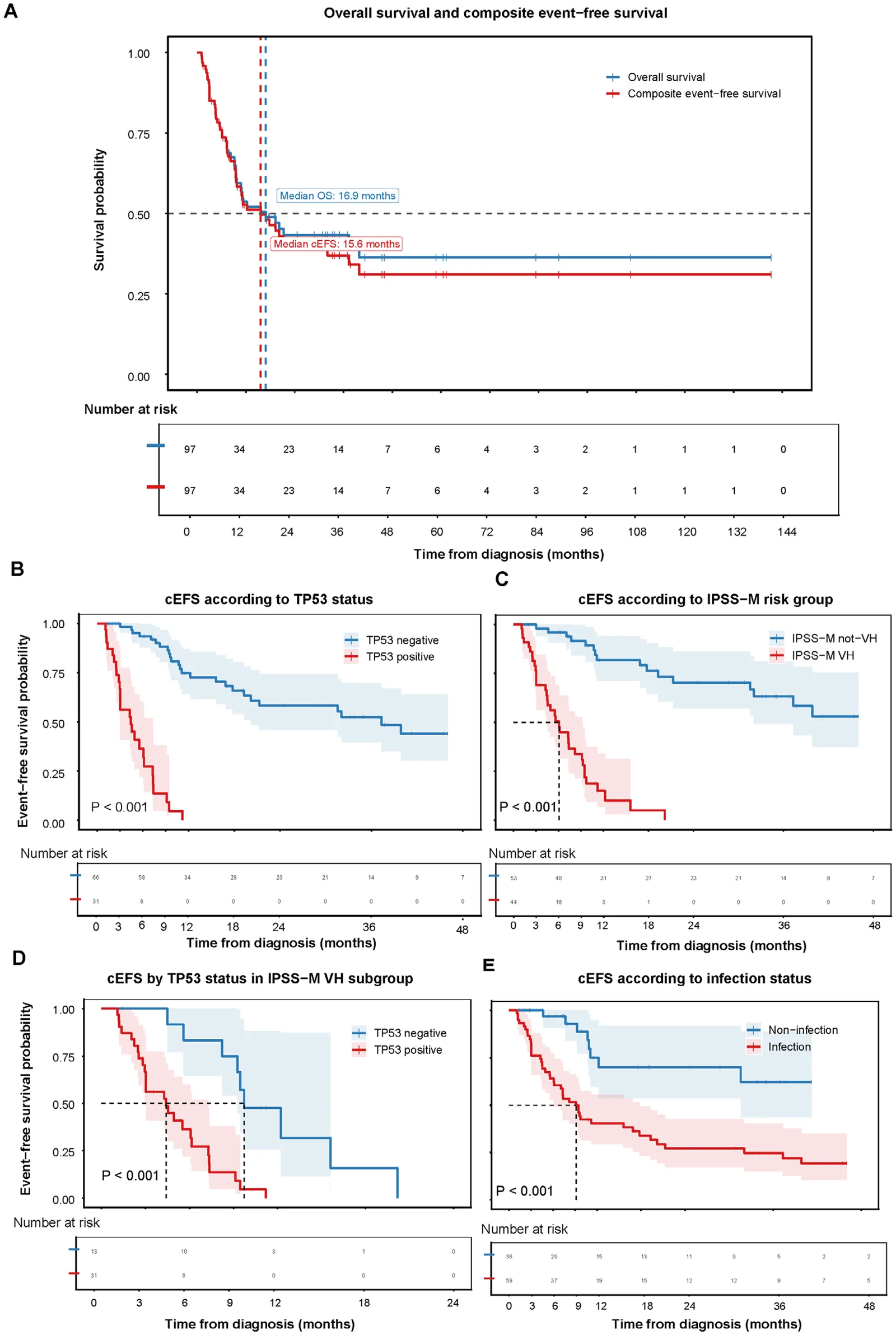
Overall survival and composite event-free survival analyses. **(A)** Kaplan–Meier curves for overall survival (OS) and composite event-free survival (cEFS) in the overall cohort. OS counted death from any cause; cEFS counted the first infection-related death or AML transformation. AML transformation ended event-free follow-up for cEFS but did not itself end OS follow-up. **(B)** cEFS by bi-TP53 status. **(C)** cEFS by IPSS-M very-high-risk status. **(D)** cEFS by bi-TP53 status within the IPSS-M very-high-risk subgroup. In panels B and D, TP53-positive and TP53-negative labels denote the bi-TP53 and non-bi-TP53 groups, respectively; non-bi-TP53 does not necessarily mean TP53 wild type. **(E)** Exploratory cEFS comparison according to infection documented during follow-up. Infection status was not a baseline exposure, and the comparison is susceptible to time-related bias and overlap with the endpoint. Time is measured from MDS diagnosis, rather than cohort identification.

### Cox regression analysis for composite event-free survival

In univariable Cox regression analysis, age, bi-*TP53* status, CD4, CD8, CRP, FN, infection status, IPSS-M VH, IPSS-R level, and NK cells were associated with cEFS. Bi-*TP53* alterations were associated with a higher cEFS event risk (HR = 15.91, 95% CI: 7.7085–32.8488; P<0.001), as was IPSS-M VH (HR = 12.11, 95% CI: 5.7775–25.3642; P<0.001). Infection status was also associated with cEFS in univariable analysis (HR = 3.98, 95% CI: 1.8683–8.4670; P<0.001), as was FN (HR = 2.09, 95% CI: 1.1860–3.6668; P = 0.011).

In the multivariable Cox model, age, bi-*TP53* status, and IPSS-M VH remained statistically associated with cEFS. Each 1-year increase in age was associated with higher cEFS event risk (HR = 1.05, 95% CI: 1.0147–1.0903; P = 0.006). Bi-*TP53* alterations remained associated with cEFS event risk (HR = 7.56, 95% CI: 2.7915–20.4565; P<0.001), and IPSS-M VH also remained significant (HR = 3.51, 95% CI: 1.1573–10.6191; P = 0.027) (**Table 3**).

**Table 3:** Univariable and multivariable Cox regression analyses for composite event-free survival.

Variables	Univariable			Multivariable		
	HR	95% CI	P value	HR	95% CI	P value
Age	1.05	1.0219–1.0851	0.001	1.05	1.0147–1.0903	0.006
Baseline ANC	1.16	0.9733–1.3774	0.098	—	—	—
Bi-TP53	15.91	7.7085–32.8488	<0.001	7.56	2.7915–20.4565	<0.001
Bone marrow blasts, %	1.04	0.9758–1.1051	0.234	—	—	—
CD19	1.00	0.9978–1.0054	0.407	—	—	—
CD3	1.00	0.9987–1.0001	0.076	—	—	—
CD4+ T cells	1.00	0.9956–0.9986	<0.001	1.00	0.997–1.0011	0.362
CD4/CD8 ratio	1.23	0.9408–1.6169	0.129	—	—	—
CD8	1.00	0.9951–0.9994	0.014	1.00	0.9978–1.0028	0.796
CRP	1.00	1.0008–1.0084	0.016	1.00	0.9951–1.0063	0.813
Diabetes mellitus	1.36	0.7119–2.6033	0.351	—	—	—
FN	2.09	1.186–3.6668	0.011	—	—	—
Hemoglobin	0.99	0.9782–1.005	0.216	—	—	—
Infection	3.98	1.8683–8.4670	<0.001	1.43	0.5205–3.907	0.49
IPSS-M very high	12.11	5.7775–25.3642	<0.001	3.51	1.1573–10.6191	0.027
IPSS-R level	10.42	4.8588-22.3305	<0.001	1.11	0.3790–3.2270	0.854
Absolute lymphocyte count	1.00	0.9995–1.0005	0.989	—	—	—
Number of co-mutations	0.94	0.7576–1.1681	0.58	—	—	—
NK	1.00	0.9954–0.9998	0.032	1.00	0.9985–1.0039	0.371
Platelets	1.00	0.9965–1.0031	0.893	—	—	—
Sex	0.83	0.4678–1.4576	0.509	—	—	—
Serum ferritin	1.00	0.9998–1.0012	0.142	—	—	—

HR, hazard ratio; CI, confidence interval; ANC, absolute neutrophil count; CTCAE, Common Terminology Criteria for Adverse Events; FN, febrile neutropenia; NA, not applicable/not included in the multivariable model. The adjusted model included age, bi-TP53 status, CD4+ and CD8+ T-cell counts, CRP, infection status, IPSS-M very-high-risk status, IPSS-R level, and NK-cell count. Febrile neutropenia was excluded after assessment of its correlation with baseline ANC. Infection status was determined during follow-up and was analyzed as a fixed covariate; related estimates are exploratory.

### Firth logistic regression for infection-related death among infected patients

Among infected patients, Firth penalized logistic regression was used to evaluate factors associated with infection-related death. In univariable analysis, IPSS-R level, IPSS-M VH, bi-*TP53* alterations, MDS treatment category, and average infection duration were associated with infection-related death. The OR was 16.6923 for IPSS-R level (95% CI: 3.3336–83.5842; P<0.001), 16.7280 for IPSS-M VH (95% CI: 3.8187–73.2771; P<0.001), 33.0741 for bi-*TP53* alterations (95% CI: 8.0248–136.3134; P<0.001), and 1.1435 for average infection duration (95% CI: 1.0603–1.2332; P<0.001).

In the multivariable Firth logistic model, bi-*TP53* alterations remained statistically associated with infection-related death (OR = 7.52, 95% CI: 1.1454–49.3702; P = 0.036). In univariable analysis, IPSS-R level, IPSS-M very-high-risk status, bi-*TP53* alterations, MDS-directed treatment, and average infection duration were associated with infection-related death. MDS-directed treatment did not meet the prespecified P < 0.01 entry criterion (P = 0.034) and was therefore not included in the multivariable Firth model. IPSS-R level, IPSS-M VH, and average infection duration were not statistically significant in the multivariable model. Overlap among IPSS-R, IPSS-M VH, cytogenetic risk, and bi-*TP53* status limits interpretation of the adjusted estimate (**Table 4**).

**Table 4:** Univariable and multivariable Firth logistic regression analyses for infection-related death among infected patients.

Variables	Univariable analysis			Multivariable analysis		
	OR	95% CI	P value	OR	95% CI	P value
Male sex	0.3647	0.121–1.0996	0.073	—	—	—
IPSS-R risk level	16.6923	3.3336–83.5842	<0.001	1.7953	0.2093–15.3979	0.594
IPSS-M very high risk	16.728	3.8187–73.2771	<0.001	1.6761	0.219–12.8297	0.619
Diabetes mellitus	1.7771	0.5478–5.7651	0.338	—	—	—
MDS-directed treatment	0.2401	0.0641–0.8985	0.034	—	—	—
FN	0.8892	0.3208–2.4649	0.822	—	—	—
Bi-TP53	33.0741	8.0248–136.3134	<0.001	7.52	1.1454–49.3702	0.036
Antibiotic level	0.4419	0.0653–2.9904	0.403	—	—	—
Recurrent infection	1.0163	0.3595–2.8732	0.976	—	—	—
Age	1.0347	0.9806–1.0918	0.214	—	—	—
Bone marrow blasts, %	0.9889	0.8904–1.0984	0.835	—	—	—
Baseline ANC	1.182	0.7881–1.773	0.419	—	—	—
Hemoglobin	0.9763	0.9502–1.0032	0.083	—	—	—
Platelet count	0.9979	0.9913–1.0045	0.533	—	—	—
Serum ferritin	1.0006	0.9991–1.002	0.456	—	—	—
CRP	1.0016	0.994–1.0093	0.683	—	—	—
CD3	0.9995	0.9981–1.0009	0.494	—	—	—
CD4	0.9968	0.9936–1	0.053	—	—	—
CD8	0.9963	0.9919–1.0007	0.1	—	—	—
CD4/CD8 ratio	1.4042	0.8365–2.3573	0.199	—	—	—
CD19	1.0071	0.9982–1.0161	0.116	—	—	—
NK	0.9983	0.9938–1.0028	0.454	—	—	—
lymphocyte	1.0006	0.9997–1.0015	0.184	—	—	—
Average infection duration	1.1435	1.0603–1.2332	<0.001	1.0418	0.9692–1.1198	0.267

OR, odds ratio; CI, confidence interval; IPSS-R, Revised International Prognostic Scoring System; IPSS-M, Molecular International Prognostic Scoring System; MDS, myelodysplastic syndrome; FN, febrile neutropenia; ANC, absolute neutrophil count; CRP, C-reactive protein. Dash indicates not included in the multivariable model. The adjusted model included IPSS-R level, IPSS-M very-high-risk status, bi-TP53 status, and average infection duration. Variables with univariable P < 0.01 were included in the multivariable Firth logistic regression model.

## Discussion

In this cohort of hospitalized patients with higher-risk MDS, bi-*TP53* alterations were associated with greater infectious burden and infection-related mortality. Patients with bi-*TP53* alterations had more frequent early and recurrent infections, greater anti-infective treatment escalation, and lower baseline T-cell and NK-cell counts. Comparable baseline neutrophil counts, hemoglobin levels, and platelet counts do not establish independence from neutropenia or other confounding factors.

Our findings extend the established adverse clinical spectrum of bi-*TP53* MDS. Bernard et al. demonstrated that multi-hit *TP53* inactivation is a powerful adverse predictor of genomic instability, complex karyotype, AML transformation, and poor survival in MDS (Bernard et al., 2020). Our data suggest that, beyond leukemic progression and treatment resistance, bi-*TP53* alterations may also be closely related to severe infectious complications and infection-related mortality. Notably, infection-related death represented a major lethal event in patients with bi-*TP53* alterations, raising the possibility that fatal infections may contribute to poor outcomes before overt AML transformation in this vulnerable subgroup. This interpretation does not diminish the central role of leukemic progression in bi-*TP53* disease, but highlights infection-related mortality as an important proximal cause of death.

Our results are consistent with previous studies linking *TP53*-altered myeloid disease to infectious complications, while further refining this association in higher-risk MDS. Chen et al. reported that pulmonary infection associated with immune dysfunction was linked to poor prognosis in MDS patients with *TP53* abnormalities, but their study focused primarily on pulmonary infection and did not stratify patients by biallelic *TP53* status (Chen et al., 2023). Marvin-Peek et al. showed that *TP53*-mutated MDS/AML was associated with increased infections, bacterial pneumonia, invasive fungal infection, reduced transplantation rates, and more infection-related deaths (Marvin-Peek et al., 2023). However, that cohort included both MDS and AML patients and was partly shaped by transplantation-related decision-making, where disease stage, treatment intensity, and transplant-related immune suppression may confound infection risk. Compared with these studies, our analysis has several distinctive features. First, we focused specifically on a strictly defined higher-risk MDS cohort, thereby reducing heterogeneity related to disease stage and diagnostic category. Second, *TP53* status was refined to biallelic alterations rather than broadly defined *TP53* abnormality or mutation status. Third, we evaluated a broader spectrum of clinically relevant infection-related outcomes, including early infection, recurrent infection, infection incidence density, antibiotic escalation, infection severity, and infection-related mortality. Finally, the integration of baseline lymphocyte subset analysis allowed us to link infection burden with a measurable cellular immune phenotype.

Lower circulating T-cell and NK-cell counts may be relevant to the infectious burden associated with bi-*TP53* alterations. Infection risk in MDS also depends on neutropenia, treatment exposure, comorbidities, and disease-related immune abnormalities (Toma et al., 2012; Houtman et al., 2025). In our cohort, lower baseline CD3+, CD4+, and CD8+ T-cell counts and NK-cell counts were associated with infection occurrence. These counts were also lower in patients with bi-*TP53* alterations. Baseline measurements describe cell abundance but do not directly measure immune function or establish mediation of infection risk. Previous work reported an immunosuppressive phenotype in *TP53*-mutated MDS and secondary AML (Sallman et al., 2020). Our findings are compatible with that observation, but the underlying mechanisms and temporal relationships require prospective investigation.

IPSS-M very-high-risk status was associated with infection occurrence in univariable analysis, but not with infection-related mortality in the multivariable model. Bi-*TP53* status remained associated with infection-related death. However, all 31 patients with bi-*TP53* alterations were classified as IPSS-M very high risk (**Table 2**). This overlap, the small sample, and the wide confidence interval limit interpretation of the adjusted estimate and any claim of independent prognostic value.

Treatment strategy and baseline ferritin should be interpreted as contextual rather than primary drivers of infection risk. Infection incidence did not differ substantially among patients receiving best supportive care, hypomethylating agent monotherapy, or HMA-based combination therapy, and the higher infection-related mortality observed in patients receiving best supportive care may reflect differences in baseline vulnerability, clinical frailty, and treatment selection (Toma et al., 2012). These treatment comparisons may have been influenced by confounding by indication. Anti-infective escalation and antifungal therapy were assessed after infection onset and therefore reflected infection severity or treatment intensity rather than baseline exposure. Baseline ferritin was elevated in both infected patients and those with bi-*TP53* alterations, raising the possibility that bi-*TP53* status may be linked to higher iron or inflammatory burden, which in turn could contribute to increased infection susceptibility (Toma et al., 2012; Wong et al., 2018; Weber et al., 2021). While our study cannot establish a causal relationship between bi-*TP53* alterations, ferritin elevation, and infection risk, this observation highlights a potential mechanistic connection that warrants further investigation in prospective studies.

These findings may have practical implications for infection surveillance in higher-risk MDS. Rather than relying solely on baseline neutrophil count or conventional cytopenia parameters, infection risk assessment may need to incorporate molecular risk, particularly bi-*TP53* status, together with cellular immune profiling. Such an approach may help identify patients who require closer monitoring, earlier diagnostic evaluation for occult infection, and more individualized supportive-care strategies. Although transplant recipients were excluded from our cohort, the early and often fatal infectious burden observed in bi-*TP53* patients suggests that timely transplant referral before severe infectious complications occur may merit prospective evaluation in carefully selected transplant-eligible patients (Nawas and Kosuri, 2024). However, universal antibacterial or antifungal prophylaxis cannot be recommended based on these retrospective data. Prophylactic or pre-emptive strategies in bi-*TP53* MDS should therefore be evaluated prospectively and tailored according to established high-risk settings, local pathogen distribution, antimicrobial resistance patterns, treatment exposure, neutropenia duration, and immune-cell dynamics (Taplitz et al., 2018; Wang et al., 2023; Wu et al., 2024).

Several limitations should be acknowledged. First, this single-center retrospective study included 97 hospitalized patients, limiting generalizability. Hospital-based inclusion and exclusion of incomplete records may introduce selection bias. For patients diagnosed earlier, inclusion may also depend on survival to hospitalization during the identification period. Second, retrospective ascertainment of infections, antimicrobial exposure, and causes of death may introduce information bias. Limited microbiological confirmation restricted comprehensive pathogen-specific analyses and evaluation of same-organism recurrence. Third, IPSS-R, IPSS-M, cytogenetic risk, and bi-*TP53* status share prognostic information. Despite Firth penalization, the limited number of events leaves residual confounding and model instability. Fourth, infection status and average infection duration were determined during follow-up. Analyses involving these variables are susceptible to time-related bias and reverse causation. Infection-related death is also a component of cEFS, creating overlap between infection status and the endpoint. Finally, the absence of longitudinal immune profiling and functional assays limits mechanistic interpretation. Multiple exploratory endpoints and subgroup analyses were not adjusted for multiplicity; therefore, the reported P values are nominal and the findings are hypothesis-generating. The small sample size, limited number of events, retrospective hospitalized cohort, and overlap among bi-*TP53* status, IPSS-M/IPSS-R risk, and cytogenetic risk further limit causal and independent interpretation.

## Conclusion

In this single-center retrospective cohort of hospitalized patients with higher-risk MDS, bi-*TP53* alterations were associated with greater infectious burden and higher infection-related mortality. Patients with bi-*TP53* alterations also had lower circulating T-cell and NK-cell counts. These associations do not establish that *TP53* alterations cause immune dysfunction or infection. Prospective multicenter studies should evaluate whether *TP53* allelic status and immune-cell measurements improve infection risk assessment beyond established clinical factors.

## Data Availability

The raw data supporting the conclusions of this article will be made available by the authors, without undue reservation.
