# Supplementary material for: Infectious burden and cellular immune impairment in higher-risk myelodysplastic syndromes with biallelic TP53 alterations
Source: Front Cell Infect Microbiol. 2026 Oct 14;16:1908881. doi: 10.3389/fcimb.2026.1908881 (PMC13713890; doi:10.3389/fcimb.2026.1908881)
Supplement: Supplementary file 1 [file Table1.docx]

**Supplementary Table S1. Distribution of infection sites and microbiological categories among infected patients.**

| **Characteristic** | **No. of infection episodes** | **% of all infection episodes** |
| --- | --- | --- |
| **Infection site** |  |  |
| Pneumonia | 61 | 41.8 |
| Bloodstream infection | 6 | 4.1 |
| Urinary tract infection | 5 | 3.4 |
| Gastrointestinal infection | 7 | 4.8 |
| Skin/soft tissue infection | 9 | 6.2 |
| Unknown site | 58 | 39.7 |
| **Microbiological category** |  |  |
| Bacterial infection | 39 | 26.7 |
| Viral infection | 7 | 4.8 |
| Fungal infection | 6 | 4.1 |
| Mixed infection | 4 | 2.7 |
| Unidentified pathogen | 90 | 61.6 |

Data are presented at the infection-episode level. A total of 146 infection episodes occurred in 59 infected patients. Percentages were calculated using 146 infection episodes as the denominator. Documented microbiological evidence was available for 56 episodes; pathogens were unidentified in the remaining 90 episodes.

Abbreviations: MDS, myelodysplastic syndrome.
